# Prognostic implication of downregulated exosomal miRNAs in patients with sepsis: a cross-sectional study with bioinformatics analysis

**DOI:** 10.1186/s40560-023-00683-2

**Published:** 2023-08-03

**Authors:** Beomsu Shin, Jin Young Lee, Yunjoo Im, Hongseok Yoo, Junseon Park, Joo Sang Lee, Ki-Young Lee, Kyeongman Jeon

**Affiliations:** 1 Division of Pulmonary and Critical Care Medicine, Department of Medicine, Samsung Changwon Hospital, Sungkyunkwan University School of Medicine, Changwon, Republic of Korea; 2 Division of Pulmonary and Critical Care Medicine, Department of Medicine, Samsung Medical Center, Sungkyunkwan University School of Medicine, 81 Irwon-Ro, Gangnam-Gu, Seoul, 06351 Republic of Korea; 3 Department of Critical Care Medicine, Samsung Medical Center, Sungkyunkwan University School of Medicine, Seoul, Republic of Korea; 4 Department of Artificial Intelligence and Department of Precision Medicine, Sungkyunkwan University School of Medicine, Suwon, Republic of Korea; 5 Department of Molecular Cell Biology, Samsung Biomedical Research Institute, Sungkyunkwan University School of Medicine, Suwon, Republic of Korea; 6 Department of Health Sciences and Technology, SAIHST, Sungkyunkawan University, Seoul, Republic of Korea

**Keywords:** Sepsis, Biomarkers, Extracellular vesicles, Exosomes, MicroRNAs

## Abstract

**Background:**

Despite the understanding of sepsis-induced extracellular vesicles (EVs), such as exosomes, and their role in intercellular communication during sepsis, little is known about EV contents such as microRNA (miRNA), which modulate important cellular processes contributing to sepsis in body fluids. This study aimed to analyze the differential expression of exosomal miRNAs in plasma samples collected from sepsis patients and healthy controls, and to identify potential miRNA regulatory pathways contributing to sepsis pathogenesis.

**Methods:**

Quantitative real-time PCR-based microarrays were used to profile plasma exosomal miRNA expression levels in 135 patients with sepsis and 11 healthy controls from an ongoing prospective registry of critically ill adult patients admitted to the intensive care unit. The identified exosomal miRNAs were tested in an external validation cohort (35 sepsis patients and 10 healthy controls). And then, functional enrichment analyses of gene ontology, KEGG pathway analysis, and protein–protein interaction network and cluster analyses were performed based on the potential target genes of the grouped miRNAs. Finally, to evaluate the performance of the identified exosomal miRNAs in predicting in-hospital and 90-day mortalities of sepsis patients, receiver operating characteristic curve (ROC) and Kaplan–Meier analyses were performed.

**Results:**

Compared with healthy controls, plasma exosomes from sepsis patients showed significant changes in 25 miRNAs; eight miRNAs were upregulated and 17 downregulated. Additionally, the levels of hsa-let-7f-5p, miR-331-3p miR-301a-3p, and miR-335-5p were significantly lower in sepsis patients than in healthy controls (*p* < 0.0001). These four miRNAs were confirmed in an external validation cohort. In addition, the most common pathway for these four miRNAs were PI3K-Akt and mitogen-activated protein kinase (MAPK) signaling pathways based on the KEGG analysis. The area under the ROC of hsa-let-7f-5p, miR-331-3p, miR-301a-3p, and miR-335-5p level for in-hospital mortality was 0.913, 0.931, 0.929, and 0.957, respectively (*p* < 0.001), as confirmed in an external validation cohort. Also, the Kaplan–Meier analysis showed a significant difference in 90-day mortality between sepsis patients with high and low miR-335-5p, miR-301a-3p, hsa-let-7f-5p, and miR-331-3p levels (*p* < 0.001, log-rank test).

**Conclusion:**

Among the differentially-expressed miRNAs detected in microarrays, the top four downregulated exosomal miRNAs (hsa-let-7f-5p, miR-331-3p miR-301a-3p, and miR-335-5p) were identified as independent prognostic factors for in-hospital and 90-day mortalities among sepsis patients. Bioinformatics analysis demonstrated that these four microRNAs might provide a significant contribution to sepsis pathogenesis through PI3K-Akt and MAPK signaling pathway.

**Supplementary Information:**

The online version contains supplementary material available at 10.1186/s40560-023-00683-2.

## Background

Sepsis is a life-threatening multisystemic dysfunction caused by a dysregulated host immune response to infection [1]. Given its significant global health burden, a tremendous effort to develop specific diagnoses of sepsis, but as yet these have not been successful [2]. Hence, there is a need to recognize sepsis early and more precisely categorize patients. In this regard, research into biological markers has not been so far successful [3]. Current sepsis biomarkers, including lactate, C-reactive protein (CRP), and procalcitonin (PCT) can be used to determine organ failure and evaluate the patient’s clinical course. However, these biomarkers have limited specificity and sensitivity in elucidating the complex regulatory mechanism of sepsis [4].

Exosomes are membrane-bound extracellular vesicles released from almost all cell types and are used as diagnostic markers in several diseases, including malignancy, cardiovascular disease, and chronic lung disease [5–7]. In addition, previous studies have shown the clinical importance of exosomes in sepsis especially based on their use as biomarkers [8, 9]. Recently, we demonstrated that plasma exosomes are highly predictive of cross-sectional and longitudinal sepsis outcomes [10, 11].

Exosomes contain cargo molecules including RNA, lipids, and proteins, which they transfer to recipient cells [12]. Among them, microRNAs (miRNAs) are a class of endogenous non-coding RNAs that mediate biological processes such as cell-to-cell communication via gene regulation by binding to their target messenger RNAs [13], cell proliferation, apoptosis, and differentiation [14]. Furthermore, miRNAs are extraordinarily stable in blood and reliably detected at low concentrations with currently available analytical methods in clinical laboratories [15]. Therefore, plasma miRNAs have been proposed as sepsis biomarkers for reflecting the pathophysiological changes at early stages [16]. However, the exosomal miRNAs related to sepsis are not fully understood [17]. In the present study, we compared the expression of sepsis-associated exosomal miRNAs in plasma collected from sepsis patients and healthy controls using miRNA PCR array profiling and validated the identified exosomal miRNAs in an independent samples from sepsis patients.

## Methods

We analyzed clinical data and prospectively collected samples from the Samsung Medical Center Registry of Critical Illness (SMC RoCI), an ongoing prospective registry of critically ill adult patients from a tertiary referral center in Seoul, South Korea (Samsung Medical Center, 1989 beds, university affiliated). This cohort was initially recruited in April 2014 for the establishment of a human sample repository and discovery of new biological markers for critical illness [10]. Informed consent prior to enrollment including the research purpose, extraction of clinical data and blood specimens, and future reporting of collected data was obtained from all study participants or their legal representatives. This study was conducted according to the Declaration of Helsinki and approved by the Ethics Committee of Samsung Medical Center (IRB no. 2013-12-033).

### Study population

**Fig. 1:**
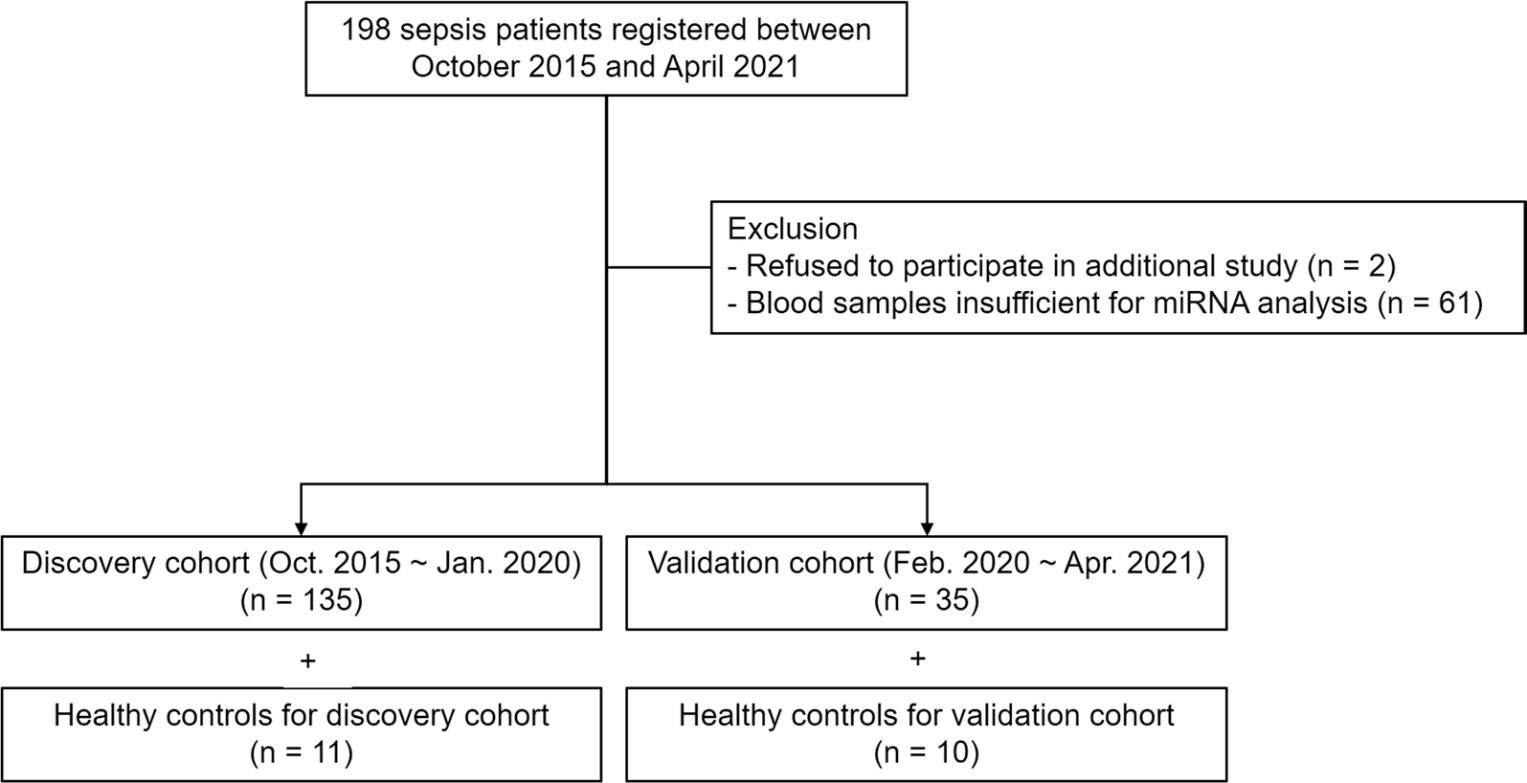
Patient flow chart

The protocols of patient enrollment and data collection for the sample repository have been previously described [10]. Briefly, critically ill adult patients (≥ 19 years old) admitted to the medical intensive care unit (ICU) were prospectively enrolled, and baseline demographics and clinical details were collected. As this is an ongoing cohort, we included a total of 135 patients with sepsis between October 2015 and January 2020 for the discovery cohort. The diagnosis of sepsis was based on the guidelines of the third International Consensus Definitions for Sepsis and Septic Shock (Sepsis-3) [1]. Since enrollment for this study began in October 2015, patients enrolled before the new definition were reclassified. In addition, 11 healthy controls (≥ 19 years) donated blood specimens (5 mL each) for research purposes. Written informed consent was obtained from all healthy volunteers. Independent data of additional critically ill patients with sepsis (*n* = 35) between February 2020 and April 2021 and healthy controls (*n* = 10) were used as external validation cohort (Fig. 1).

### Data collection

A trained study coordinator used patient hospital records to prepare a standardized case report form. Clinical data consisting of patient demographics, reasons for ICU admission, severity of illness, and laboratory data (PaO_2_/FiO_2_, lactic acid, CRP, PCT, interleukin-6 (IL-6), platelet count, albumin, total bilirubin, and creatinine levels) were obtained at enrollment. Severity of illness was assessed using the Acute Physiology and Chronic Health Evaluation II (APACHE II), Simplified Acute Physiology Score 3 (SAPS 3), and Sequential Organ Failure Assessment (SOFA) scores [18–20]. In addition to clinical data, 19 mL of whole blood were drawn from each patient within 48 h of enrollment.

### Exosomal miRNAs analysis

#### Plasma preparation

Blood samples (≥ 15 mL) were collected for plasma preparation. Plasma was prepared by drawing peripheral blood into ethylenediaminetetraacetic acid tubes, followed by centrifugation at 480 g (Eppendorf Centrifuge 5810 No. 0012529-rotor A-4-81) for 10 min at 4 °C. Several plasma aliquots from each study participant were isolated and stored at − 80 °C until further analysis. Prior to exosome isolation and RNA purification, plasma samples were pre-filtered using 0.8 µm syringe filters, followed by additional centrifugation to eliminate residual cellular debris.

#### Exosome isolation

Exosomes were isolated from plasma samples using ExoQuick Plasma prep and Exosome isolation kit (System Biosciences, CA, USA). We modified the manufacturer’s protocol for sample-optimized exosome isolation. The modified protocol was intended to avoid unnecessary sample waste and remove residual impurities. An ExoQuick reagent was used to precipitate plasma exosomes, while the ExoRNeasy mini kit (Qiagen) was used to directly purify RNA from exosomes. To isolate exosomes, 250 µL of the filtered plasma was added to 63 µL of ExoQuick (System Biosciences, CA, USA) and incubated for 30 min at 4 °C. The ExoQuick/plasma mixture was centrifuged at 1500 *g* for 30 min, followed by supernatant removal and centrifugation of the EV pellet at 1500 *g* for 5 min to remove the residual ExoQuick solution. The EV pellet was resuspended in 200 µL of phosphate-buffered saline, and the precipitated exosomes were used immediately.

#### Characterization of exosomes

To analyze cell-specific plasma exosomes, the isolated exosomes were suspended in Phosphate-Buffered Saline containing 1% heat-inactivated fetal bovine serum (FACS buffer) and rested at 37 °C for 15 min. The exosomes were then incubated with 10 µg/ml of the appropriate primary antibody [CD235a (granulocyte), CD3 (lymphocytes), CD11b (monocytes), CD4L (platelet)] plus the exosomal marker CD63. After incubation, the exosomes were washed twice with ice-cold FACS buffer and then stained with 0.25 µg of a FITC-linked secondary antibody for 30 min at 4 °C. After washing with FACS buffer three times, the exosome counts were analyzed by gating the live cell population based on forward and side scatter properties. Flow cytometry data were obtained by a FACS Canto flow cytometer using FACSDiva v8.0 software (BD Biosciences) and analysed using FlowJo v10.0.5 (Tree Star, Ashland, OR). The exosome counts were proportional to the number of fluorescently labelled exosomes, CD63 (total exosomes), [CD235a, CD63]-granulocyte-specific exosomes, [CD3, CD63]-lymphocyte-specific exosomes, [CD11b, CD63]-monocyte-specific exosomes, and [CD4L, CD63]-platelet-specific exosomes.

The concentration and size distribution of the isolated exosomes were assessed by nanoparticle tracking analysis (NTA) using a Nanosight NS300 (NanoSight Ltd., Amesbury, UK). Protein quantification of plasma-derived exosome preparations were performed using Pierce BCA Protein Assay kit (Thermo Fisher Scientific, MA, USA).

#### Total RNA isolation and quality analysis

Total RNA was extracted from plasma-isolated exosomes using the exoRNeasy Midi Kit following manufacturer’s instructions (Qiagen, Valencia, CA, USA). Briefly, the filtered samples were washed and centrifuged using Buffer XBP and XWP. The isolated exosomes were eluted in 700 µL of QIAzol reagent (QIAGEN Cat. 79,306) and spiked with RNA Spike-In Control (QIAGEN Cat. 339,390) before proceeding with the extraction. Then, 90 µL of chloroform was added to the lysate for subsequent phase separation. The upper aqueous phase containing RNA was transferred to a new collection tube, and 400 µL of the aqueous phase mixed with 800 µL of 100% ethanol; the mixture was transferred into an RNeasy MinElute spin column in a 2 mL collection tube. After centrifugation, the RNA was adhered to the column membrane, and washed with Buffer RWT and RPE; then, DNase/RNase-Free water was added and centrifuged 1 ~ 2 min at 10,000 × *g*, the collected flow-through corresponded to the exosome total RNA. RNA quantity was measured by NanoDrop 2000 (Thermo Fisher Scientific, MA, USA). RNA purity was assayed by the assessing the 260/280 nm and 260/230 nm absorbance ratios. RNA integrity was assessed using an Agilent 2100 Bioanalyzer with an RNA 6000 Pico kit (Agilent Technologies, CA, USA) (see Additional file 1: Fig. S1). RNA libraries were constructed from 3 ng of total RNA using the SMARTer smRNA-Seq Kit for Illumina (Takara Bio, Shiga, Japan).

#### Reverse transcription and microRNA expression profiling

The miRCURY LNA™ miRNA Focus PCR panel was used for miRNA profiling of exosomal miRNAs from patients with sepsis and healthy controls (QIAGEN, Cat no. 339325, YAHS-106Y, Qiagen, Hilgen, Germany) (see Additional file 1: Fig. S2). The total extracted RNA including miRNAs (10 ng/µL concentration) was first reverse transcribed into first-strand cDNA using a single miRCURY LNA miRNA PCR assay following manufacturer’s instructions (QIAGEN, Cat no. 339306). Then, 1 µL cDNA per well was mixed with SYBR Green qPCR Master Mix and placed into two 96-well PCR-array plates containing a panel of 179 miRNAs sequences. One µL cDNA was brought to a 10 µL final volume reaction for real-time PCR analysis using an Applied Biosystems Step-One Plus Real-Time PCR system. Relative amounts were calculated using the ΔΔC_T_ method. Samples without good RNA quality were excluded from statistical analysis.

Sequencing data analysis was performed using the GeneGlobe Data Analysis Center (Qiagen). GeneGlobe provides the fold-change (FC = miRNA expression of the nephrotoxicity group / miRNA expression of the non-nephrotoxicity group), FR (FR = FC, if FC ≥ 1 or FR = FC ≥ 1, if FC < 1) and *p*-value based on the Wald test for each miRNA. miRNA analysis was performed using the geNorm method to identify the best reference controls. The median miR-486-5p, miR-151a-5p, and hsa-miR-532-3p expression was used for data normalization [21]. The relative gene expression was calculated using the comparative cycle threshold (2^−ΔΔCT^) method [22].

#### Validation of differentially-expressed miRNAs by quantitative RT-PCR

Custom 96-wells Pick-&-Mix microRNA PCR plates (Qiagen) were used to validate teach miRNA candidate. qRT-PCR was performed to quantify the expression levels of candidate exosomal miRNAs using an miRCURY LNA miRNA PCR Starter Kit (Qiagen, No. 339320) and a miRCURY LNA SYBR Green PCR Kit (Qiagen, No. 339347). In addition, we used the Applied Biosystems QuantStudio 7 Flex Real-Time PCR System in a total volume of 10 μL. qRT-PCR conditions were 95 °C for 2 min, followed by 40 cycles of 95 °C for 10 s and 56 °C for 1 min, followed by melting curve analysis. Synthetic UniSp3 was analyzed as interplate calibrator and qRT-PCR control. Amplification curves were evaluated using QuantStudio Software v1.3 (Thermo Fisher Scientific, Massachusetts, USA). Quantification cycles (Cq) > 35 cycles were censored at the minimum level observed for each miRNA. cel-miR-39-3p levels were stable across samples. Relative quantification was performed using the 2^−ΔCq^ method (ΔCq = Cq_miRNA_-Cq_cel-miR-39-3p_). Expression levels were log-transformed for statistical purposes [23].

### Bioinformatics analysis

Differentially-expressed miRNAs with FR > 2.5 or FR <  − 2.5 were selected for analysis. All selected miRNAs presented no comments or comment “A” and a *p*-value < 0.05. The miRWalk platform, which provides a list of predicted miRNA target genes according to 12 different algorithms, including TargetScan, were assessed to predict the target genes of differently expressed miRNAs (see Additional file 1: Fig. S3). Then, a protein–protein interaction (PPI) network of target genes was constructed using the STRING database (http://string-db.org). The PPI network was further visualized by Cytoscape v3.9.1. In addition, we used the Gene Ontology (GO) database (http://www.geneontology.org/) for function enrichment. The most statistically significant term within a cluster was chosen to represent the cluster. Only terms with *p*-value < 0.05 and gene numbers ≥ 1.5 were considered meaningful (≥ 1 gene) [24]. The Database for Annotation, Visualization and Integrated Discovery (DAVID) Bioinformatics resource was used to conduct functional enrichment analysis and delineate the underlying biological processes and pathways of aberrantly expressed intersecting genes based on the Kyoto Encyclopedia of Genes and Genomes (KEGG) database [25].

Based on the enriched pathways of target genes, the biological functions of the grouped miRNAs were predicted by miRsystem database, which interprets seven algorithms predicting miRNA targets (DIANA, miRanda, miRBridge, PicTar, PITA, rna22, and TargetScan) and two experimentally validated databases (TarBase and miRecords). The PPI network was established using the STRING database to annotate the functional interaction between target genes and other genes. Active interaction sources, including text mining, experiments, databases, and co-expression as well as species limited to *Homo sapiens* and an interaction score > 0.7 were applied to construct the PPI network, which showed the physical and functional interactions between genes [26]. We selected gene pairs with a total score > 0.9 for PPI network construction [27]. Then, the Cytoscape software was applied to merge the indirect PPI with driver genes’ PPI to discover interconnected and intersected functional modules and target the core genes. Eventually, we analyzed the interactions between differential expression of exosomal miRNAs and well-known sepsis mechanisms.

### Statistical analysis

Clinical data is presented as numbers (percentages) for categorical variables, and as the median and interquartile range (IQR, 25th–75th percentiles) for continuous variables. Relative miRNA levels were obtained using 2DC_T_ (2C_T_ of reference miRNAs–C_T_ of miRNA of interest) for each miRNA. The *p*-value of differential miRNA expression was calculated based on Poisson’s distribution [28] and the *p*-value threshold was determined by the false discovery rate [29]. Receiver operating characteristic (ROC) analysis was performed, and the area under the curve (AUC) reported to evaluate the performance of miRNAs in predicting in-hospital mortality of sepsis patients. In addition, patients were reclassified into two groups; high and low miRNA levels according to each optimal cutoff level calculated by Youden’s index [30]. Kaplan–Meier equation was used to determine the 90-day mortality curves according to each miRNA levels, which were then compared by the log-rank test. All tests were two-sided, and a *p*-value < 0.05 was considered statistically significant.

## Results

### Baseline characteristics of the study population

The baseline characteristics of study subjects on ICU admission are presented in Table 1. The median age was 66 years (58–74 years) and 67% were male. A total of 59 (44%) patients required mechanical ventilation and 70 (52%) patients needed vasopressor support. The median SAPS3 score on ICU admission was 55 (47–63); the median APACHE II and SOFA scores were 24 (20–30) and 9 (7–11), respectively. The interval between ICU admission to blood sampling was 30 (21–37) hours. The median serum CRP, PCT, and IL-6 were 12.5 (5.9–24.8) mg/dL, 6.06 (0.85–25.18) ng/mL, and 228 (65–807) pg/mL, respectively.

**Table 1:** Demographic and clinical characteristics of study participants (*N* = 135)

	Number (%) of patients or median (IQR)
Age, years	66 (58–74)
Sex, male	91 (67.4)
BMI, kg/m^2^	22.9 (20.6–26.0)
*Comorbidity*	
Malignancy	66 (48.9)
Diabetes	41 (30.4)
Chronic obstructive pulmonary disease	16 (11.9)
Chronic kidney disease	10 (7.4)
Cerebrovascular disease	8 (5.9)
Chronic liver disease	8 (5.9)
Congestive heart failure	7 (5.2)
Connective tissue disease	2 (1.5)
*Clinical status at ICU admission*	
Need for mechanic ventilation	59 (43.7)
Need for vasopressor support	70 (51.9)
*Severity of illness*	
SAPS3 score	55 (47–63)
APACHE II score	24 (20–30)
SOFA score	9 (7–11)
*Laboratory findings*	
CRP, mg/dL	12.5 (5.9–24.8)
Lactic acid, mmol/L	2.9 (2.0–4.4)
Procalcitonin, ng/mL	6.06 (0.85–25.18)
IL-6, pg/mL	228 (65–807)
Platelet count, 10^3^/μL	135 (59–214)
Albumin, g/dL	2.9 (2.6–3.2)
Total bilirubin, mg/dL	1.1 (0.7–2.5)
Creatinine, mg/dL	1.3 (0.8–1.9)

*IQR* interquartile range, *BMI* body mass index, *ICU* intensive care unit, *SAPS 3* simplified acute physiology score 3, *APACHE II* acute physiology, and chronic health evaluation II, *SOFA* sequential organ failure assessment, *CRP* C-reactive protein, *IL-6* interleukin-6

### Characterization of exosomes

The analysis of flow cytometry experiments demonstrated that a significant portion of vesicles was positive for CD63 and CD11b in sepsis patients compared to healthy volunteers, suggesting an exosomal and monocyte origin for these vesicles. Taken together, our results strongly suggest that the major component of our vesicle pool was composed of monocyte-derived exosomes (Fig. S4, see Additional file 1).

The levels of exosomal protein isolates from 1 mL, 2.5 mL, and 5 mL of plasma were 663 (± 500) µg, 1440 (± 204) µg, and 2270 (± 182) µg, respectively. According to the amount of plasma, the levels of exosomal RNA were 2446 (± 770) ng in 1 mL, 3010 (± 780) ng in 2.5 mL, and 5779 (± 1020) ng in 5 mL. The RNA purity was determined spectrophotometrically by means of the 260/280 nm and 260/230 nm absorbance ratios. The mean of OD260/280 was 2.04 (± 0.07) (see Additional file 2). As shown in the additional file 2, the ratios were greater than or equal to 2.0 in most samples, which indicate a high-quality level of RNA. Bioanalyzer data revealed that plasma-derived exosomes contain a broad range of RNA sizes including a high amount of small RNAs (25–200 nucleotides). Among the small RNAs, transfer RNA (18.7%) and miRNAs (14.7%) were the most abundant. Other small exosomal RNAs included ling noncoding RNA (10.5%), Y RNA (4.8%), and ribosomal RNAs (3.9%).

### Exosomal miRNA profiling

**Fig. 2:**
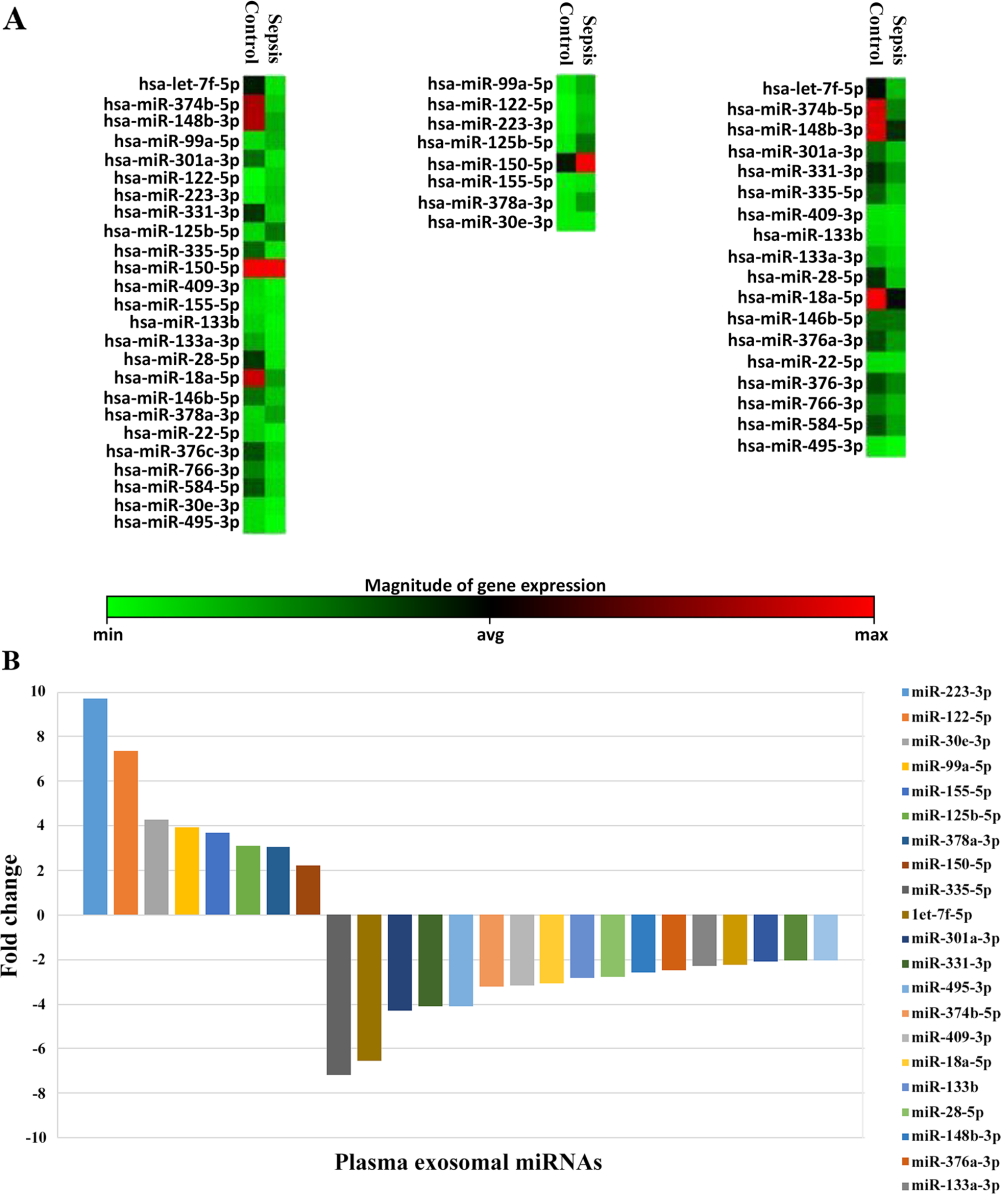
miRNA expression patterns (**A**) and fold-change in plasma exosomal miRNAs (**B**) in patients with sepsis. Compared to healthy controls, eight miRNAs were significantly elevated and 17 miRNAs significantly decreased in patients with sepsis

To analyze the miRNA content of plasma-derived exosomes, we performed miRNA profiling analysis in patients with sepsis and compared the results with those of healthy individuals; 179 miRNAs were differentially expressed compared with the controls. We found eight upregulated miRNAs (miR-223-3p, miR-122-5p, miR-30e-3p, miR-99a-5p, miR-155-5p, miR-125b-5p, miR-378a-3p, and miR-150-5p) and 17 downregulated miRNAs (miR-335-5p, hsa-let-7f-5p, miR-301a-3p, miR-331-3p, miR-495-3p, miR-374b-5p, miR-409-3p, miR-18a-5p, miR-133b, miR-28-5p, miR-148b-3p, miR-376c-3p, miR-133a-3p, miR-584-5p, miR-766-3p, miR-22-5p, and miR-376c-3p; Fig. 2).

### Validation of differentially-expressed miRNAs

**Fig. 3:**
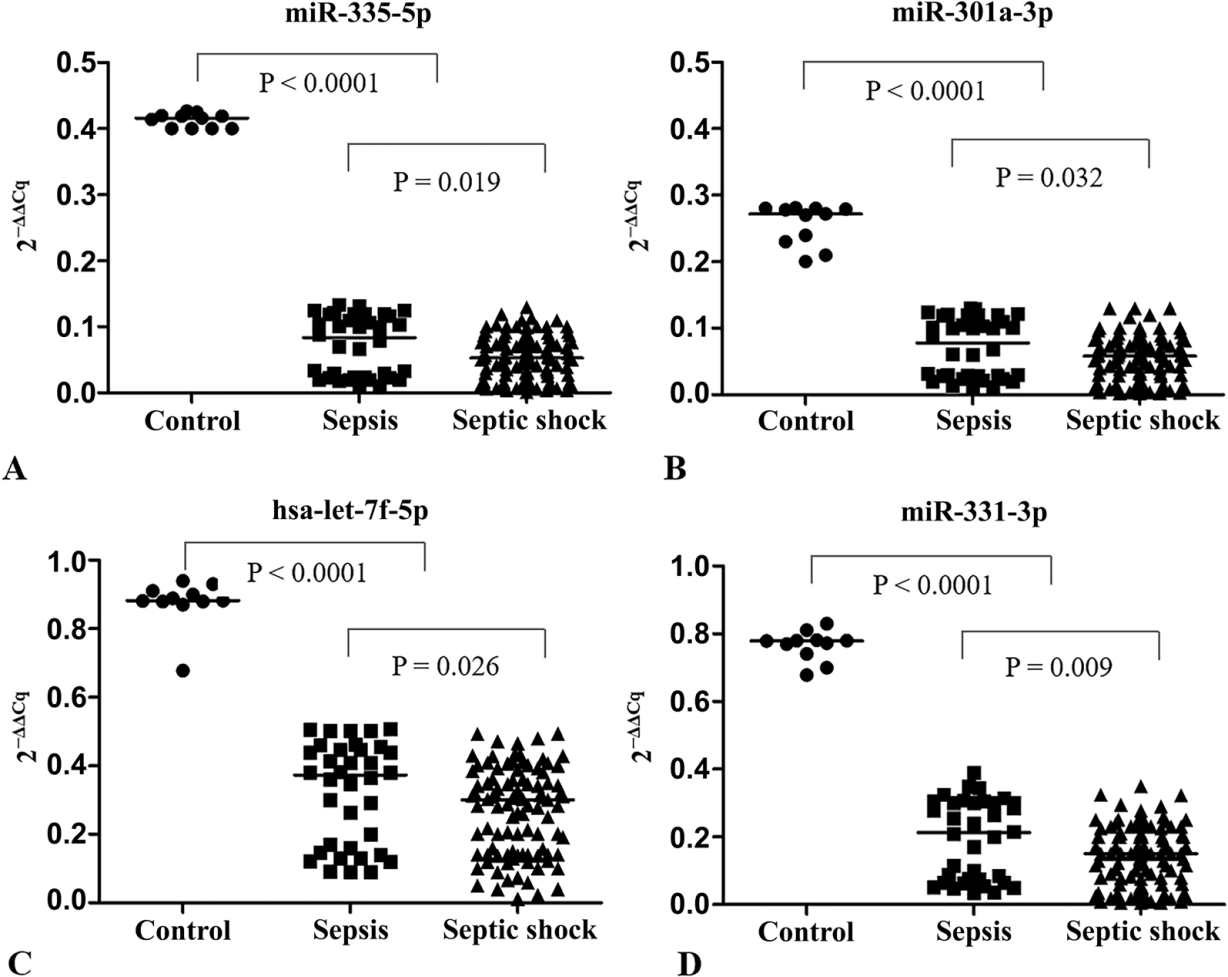
Quantitative RT-PCR validation for four differentially-expressed miRNAs in patients with sepsis (*n* = 36) and septic shock (*n* = 99), and healthy controls (*n* = 11)

We identified 25 differentially-expressed miRNAs and selected the top four downregulated exosomal miRNAs for qRT-PCR validation: hsa-let-7f-5p, miR-335-5p, miR-331-3p, and miR-301a-3p, in accordance with the microarray screening results. As shown in Fig. 3, miR-335-5p, miR-301a-3p, hsa-let-7f-5p, and miR-331-3p levels were significantly decreased in sepsis patients compared with healthy controls (*p* < 0.0001). In addition, levels of the top four exosomal miRNAs were significantly decreased in septic shock compared to sepsis (Fig. 3).

In the external validation cohort, we tested another 35 patients with sepsis and five healthy controls (see Additional file 1: Table S1) for analysis of differences in the top four downregulated exosomal miRNAs. Compared with healthy controls, the selected miRNAs were downregulated in sepsis (*p* < 0.0001; see Additional file 1: Fig. S5).

### Bioinformatics analysis

#### KEGG pathways for grouped miRNAs

**Fig. 4:**
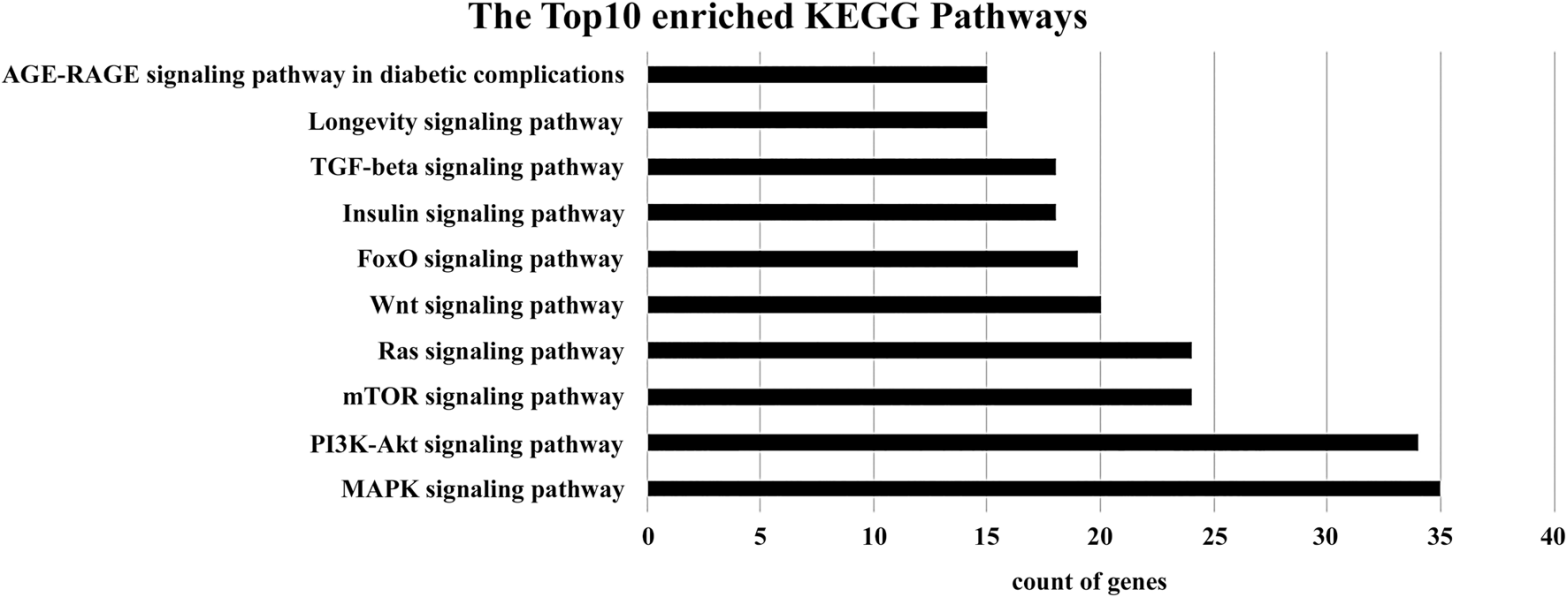
KEGG pathway analysis of four downregulated exosomal miRNAs in sepsis

KEGG pathway analysis revealed that the target genes of these four miRNAs mainly belonged to 10 signaling pathways: mitogen-activated protein kinase (MAPK), phosphatidylinositol 3-kinase/protein kinase B (PI3K-Akt), mammalian target of rapamycin (mTOR), Ras, Wnt, forkhead box protein O (FoxO), insulin, transforming growth factor-β (TGF-β), longevity regulating, and advanced glycation end products/receptor for advanced glycation end products (AGE-RAGE) (Fig. 4). Additionally, the most common pathway for hsa-let-7f-5p, miR-331, miR-301a, and miR-335 was PI3K-Akt and MAPK signaling pathway (Table 2).

**Table 2:** GO enrichment and KEGG pathway analysis

miRNAs	Enriched KEGG pathways	Count of genes
*1. has-let-7f-5p*		
hsa04151	PI3K-Akt signaling pathway	39
hsa04010	MAPK signaling pathway	39
hsa04150	mTOR signaling pathway	27
*2. miR-331-3p*		
hsa04010	MAPK signaling pathway	25
hsa04151	PI3K-Akt signaling pathway	14
hsa04014	Ras signaling pathway	11
*3. miR-301a-3p*		
hsa04010	MAPK signaling pathway	32
hsa04014	Ras signaling pathway	25
hsa04068	FoxO signaling pathway	24
*4. miR-335-5p*		
hsa04010	MAPK signaling pathway	33
hsa04151	cAMP signaling pathway	10
hsa04068	FoxO signaling pathway	9

*GO* Gene Ontology, *KEGG* Kyoto Encyclopedia of Genes and Genomes

#### GO enrichment for identifying potential target genes of the grouped miRNAs

The potential target genes of these four miRNAs were predicted by miRsystem database. A total of 1817 target genes for these four differentially-expressed miRNAs were predicted and used for GO function enrichment. The analysis of biological processes indicated that the selected miRNAs were mainly involved in cellular processes, biological regulation, and metabolic processes. With respect to molecular functions, it included binding, protein binding, and ion binding. Finally, at the cellular level, the selected miRNAs were related to cellular anatomical entity, intracellular, and organelles (see Additional file 1: Fig. S6).

#### PPI networks

To identify the core genes and interaction between genes from common KEGG pathways, we mapped 405 target genes of the grouped miRNAs to the STRING database. The PPI network consisted of 1224 nodes and 2429 edges. Enrichment pathway analysis showed that the genes were related to cancer pathways, PI3K-Akt signaling, and MAPK signaling (see Additional file 1: Fig. S7).

#### Cluster analysis of predicted genes

**Fig. 5:**
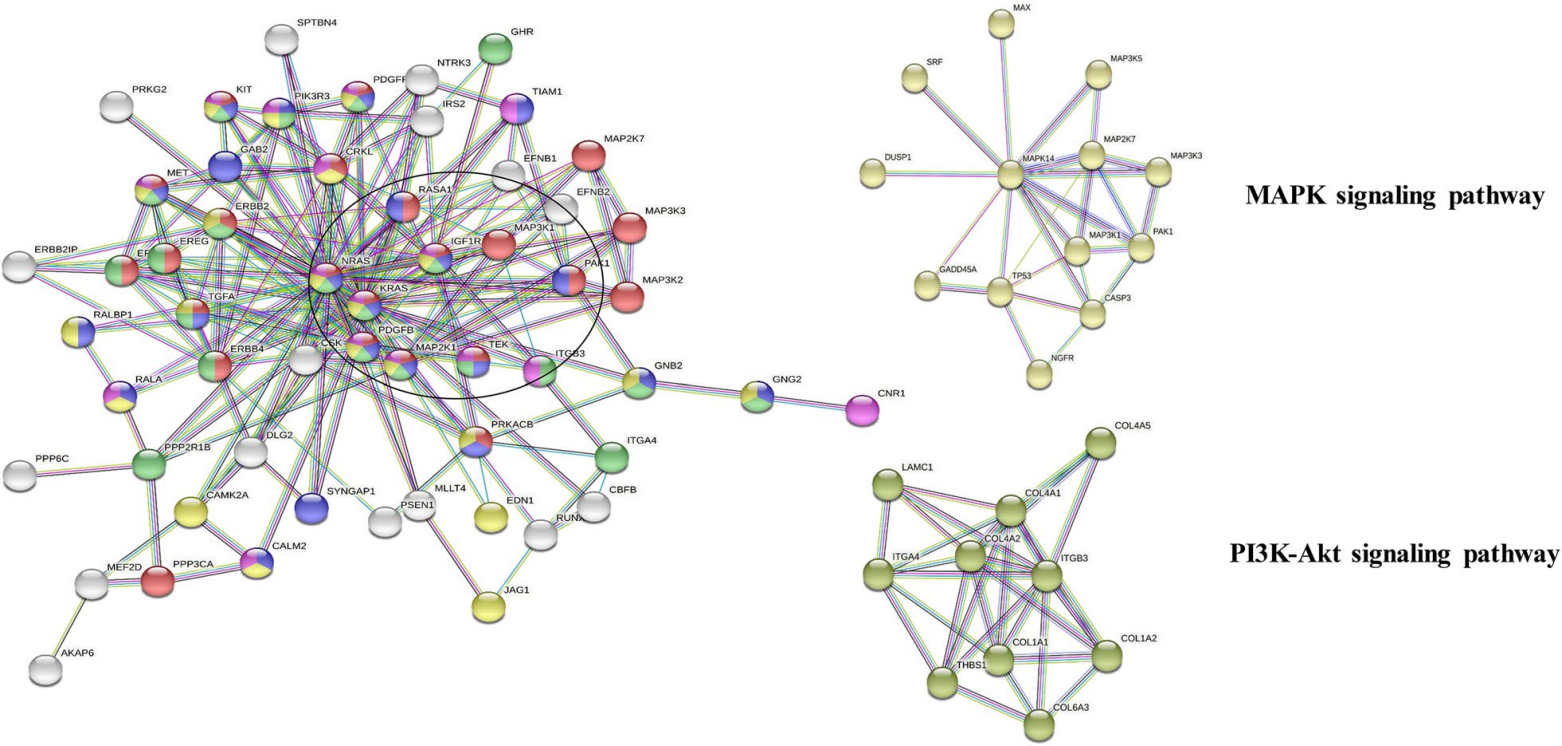
Merged protein–protein interaction networks. Dots represent genes and lines represent interactions

The cluster analysis of the predicted 405 genes was performed to discover groups of correlated genes potentially associated to disease or conditions. Finally, the results of the top 2 KEGG pathways and PPI networks were integrated as shown in Fig. 5.

### Predictive value for in-hospital and 90-day mortalities

**Fig. 6:**
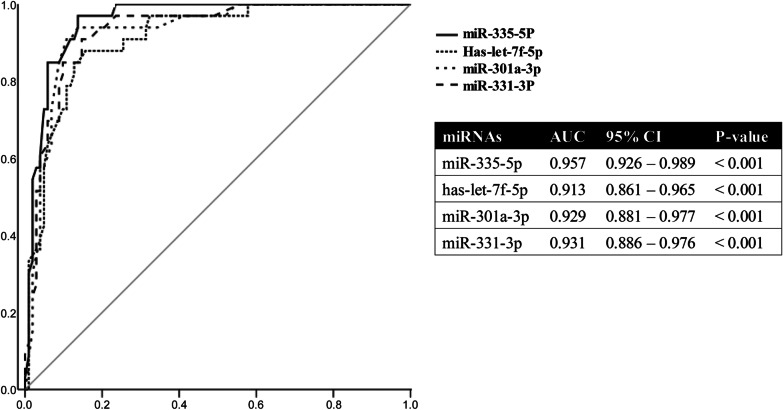
Receiver operating characteristic curves of the predictive value of four miRNAs for in-hospital mortality in patients with sepsis

**Fig. 7:**
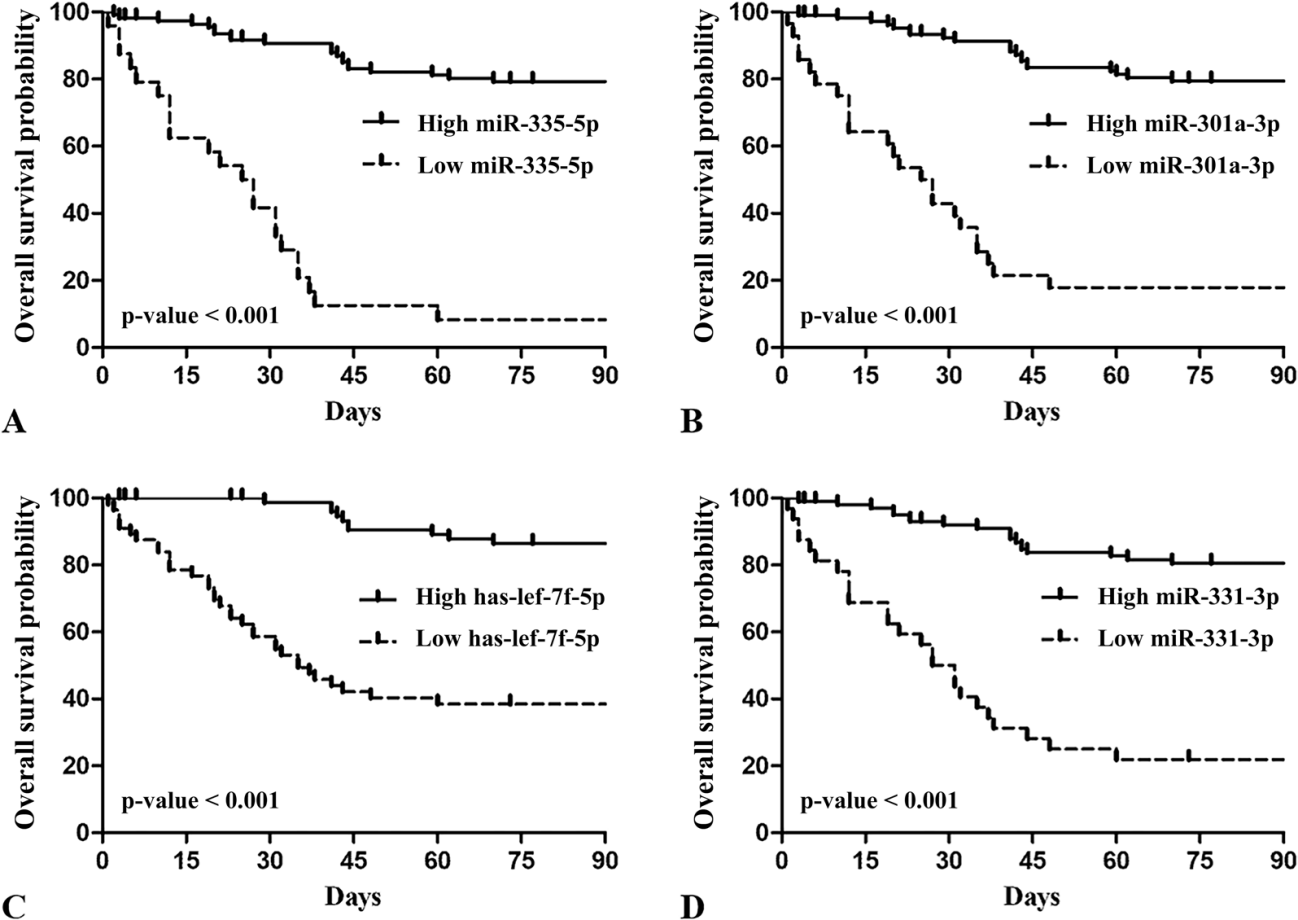
Kaplan–Meier survival estimation of sepsis patients with high and low miRNAs (**A**, miR-335-5p; **B**, miR-301a-3p; **C**, has-lef-7f-5p; **D**, miR-331-3p) for 90-day mortality

The results of ROC analysis and AUCs of the four selected miRNAs are shown in Fig. 6. The AUC of hsa-let-7f-5p, miR-335-5p, miR-331-3p, and miR-301a-3p level for in-hospital mortality was 0.913, 0.957, 0.931, and 0.929, respectively (*p* < 0.001). In the external validation cohort, their AUCs for in-hospital mortality were 0.920, 0.902, 0.907, and 0.914, respectively (*p* < 0.001) (see Additional file 1: Fig. S8). Furthermore, Kaplan–Meier survival estimation demonstrated a significant difference in 90-day survival between patients with high and low miR-335-5p, miR-301a-3p, hsa-let-7f-5p, and miR-331-3p levels, respectively (all *p* < 0.001, log-rank test) (Fig. 7).

## Discussion

We evaluated differentially-expressed miRNAs between sepsis patients and healthy controls based on the miRNA profile of plasma exosomes from patients with sepsis. A total of eight upregulated and 17 downregulated miRNAs were identified in sepsis patients. Among the differentially-expressed miRNAs detected in microarrays, the top four downregulated exosomal miRNAs were validated using qRT-PCR (miR-335-5p, miR-301a-3p, hsa-let-7f-5p, and miR-331-3p), and identified as prognostic factors for in-hospital and 90-day mortalities among sepsis patients. Based on the KEGG pathway analysis, these four miRNAs might provide a significant contribution to sepsis pathogenesis through MAPK and PI3K-Akt signaling pathway.

Sepsis is a complex syndrome that involves multiple organs and tissues; current biomarkers cannot detect its early phase [31]. To overcome these limitations, several studies investigated the pro-inflammatory role of miRNAs in sepsis [32]. Moreover, miRNA profiling has shown their potential as biomarker candidates in sepsis [33]. The miRNAs identified in the present study were evaluated in previous sepsis-related studies (Table 3). Furthermore, functional studies investigating their role revealed the effect of miR-223-3p on immune cell differentiation. miR-223 is significantly elevated during tissue inflammation, and regulates immune cell proliferation, differentiation, and polarization [34]. Moreover, Zhu et al. reported that miR-99a-5p mediates γδ T cell activation and apoptosis, serving as antigen-presenting cells in the adaptive immune response [35]. Chaudhuri et al. reported that miR-125b-5p regulates macrophage activation via IFN regulatory factor 4 levels and serves as an inflammatory response inhibitor [36]. Finally, Nie et al. reported that miR-331-3p regulates the inflammatory response via a nucleotide-binding oligomerization domain, leucine rich repeat, and pyrin domain-containing protein (NLRP) 6, which plays an important role in apoptosis, inflammation, and immune response [37]. The present results confirm that the identified miRNAs are related to sepsis pathophysiology.

**Table 3:** Previous studies on miRNAs during sepsis

Study year	miRNA	Regulation	Sample	Case	Control
2010 [43]	miR-146a	Down	Serum	50	20
2010 [43]	miR-223	Down	Serum	50	20
2013 [44]	miR-146a	Down	Plasma	14	14
2015 [45]	miR-155	Up	Serum	60	30
2016 [46]	miR-155-5p	Up	Serum	105	35
2018 [17]	miR-125b	Up	Exosome	24	12
2018 [47]	miR-223-3p	Up	Plasma	187	186
2019 [48]	miR-223-3p	Up	Plasma	44	21
2020 [49]	miR-125b-5p	Up	Plasma	120	120

*miRNA* microRNA

Herein, we obtained actively released miRNAs of plasma exosome from 135 patients with sepsis and performed qRT-PCR-based microarray analysis of 179 different miRNAs to identify additional differentially-expressed miRNAs to those in previous studies. Among them, we established down-regulation of four miRNAs (miR-335-5p, miR-301a-3p, hsa-let-7f-5p, and miR-331-3p) in patients with sepsis. To better understand the interactions of target genes in the four miRNAs, KEGG analysis was performed. Our data showed that exosomal hsa-let-7f-5p, miR-335-5p, miR-331-3p, and miR-301a-3p were concentrated on 60 signaling pathways, the top two being MAPK and PI3K-Akt signaling. The signaling pathways related to these miRNAs and thus considered related to sepsis were identified. In past studies, the MAPK and PI3K-Akt pathways were associated with cell apoptosis [38]. MAPK and p38 are crucial intracellular signal transduction pathways that mediate endothelial inflammatory injury during sepsis [39]. These pro-survival pathways control the transcription of genes related to cell apoptosis, and can be activated by various inflammatory mediators and environmental stress, inducing the excessive release of pro-inflammatory cytokines, including TNF-a, IL-6 and other inducible enzymes [40]. Further, the PI3K-Akt signaling pathway was recently associated with negative signals in the hyper-inflammatory state of sepsis [41]. The FoxO and mTOR signaling pathways downstream of PI3K-Akt signaling are also related to the cell cycle [42]. Functional studies focusing on these miRNAs are required to provide additional information with potential therapeutic value.

Additionally, the Cq values in this study were significantly greater in the sepsis group than in healthy controls, indicating reduced expression, and supporting the potential of these miRNAs as sepsis biomarkers. Furthermore, we ascertained that the low expression of miR-335-5p, miR-301a-3p, hsa-let-7f-5p, and miR-331-3p in subjects with sepsis significantly correlates with in-hospital and 90-day mortalities. These miRNAs could improve risk prediction of hospital mortality beyond APACHE II (AUC of 0.721; 95% CI, 0.624–0.817) or SAPS 3 (AUC of 0.720; 95% CI, 0.617–0.823), commonly used to predict hospital mortality in clinical practice [18, 19].

Although this study provides additional information on the differentially-expressed miRNAs as biomarkers of the diagnosis and morality prediction of sepsis via molecular experiments and clinical information from a prospective cohort, our study has certain limitations that should be acknowledged. First, the study population was relatively small; thus, future bioinformatics studies are necessary to confirm our findings. In addition, we could not evaluate the dynamic profile of exosomal miRNAs during the progression of sepsis, since subsequent samples from the enrollment were not available in many patients with sepsis. Second, this study was conducted at a single referral center in Korea, which might limit the generalization of our results, especially to other institutions or ethnic groups. Third, multiple preexisting diseases, such as diabetes and malignancies, may confound the total RNA and miRNA in exosomes with pleiotropic effects. In this study, however, sepsis patients with diabetes or malignancy were also included for unbiased comparison with the general sepsis patients, and the top four downregulated exosomal miRNAs were not associated with these underlying diseases. In addition, additional analysis with sepsis patients who have no comorbidities revealed that the selected miRNAs were downregulated in sepsis compared with healthy controls (*p* < 0.0001; see Additional file 1: Fig. S9).

## Conclusions

The present study identified plasma exosomal miRNAs with dysregulated expression in patients with sepsis compared with healthy controls; the differentially-expressed miRNAs have potential as biomarkers for sepsis. Moreover, we demonstrated their potential as early prognostic tools in sepsis. Further studies are required to elucidate the mechanisms by which these miRNAs affect intercellular communication during sepsis.

### Supplementary Information

**Additional** **file** **1:** **Table** **S1**. Demographic and clinical characteristics of the validation cohort with sepsis (N = 35). **Figure** **S1.** Bioanalyzer exosomal RNA quality control data. An ANA Agilent 2100 Bioanalyzer was used to examine exosomal RNA quality (Agilent Technologies, Inc. Santa Clara, CA, USA). The RNA ladder standard (in the first lane) contains six RNA fragments ranging between 0.2–6 kb. Representative bands of our sample’s RNA (in the second lane) showing 5S (120 nt), 18S (1,900 nt), and 28S rRNA (4,700 nt). For exoRNA bands, all samples showed an obvious band in the small RNA area. **Figure** **S2.** Hierarchical clustering of exosomal miRNA expression in a selected group of 135 sepsis patients utilizing 179 differentially-expressed miRNAs. Exosomal miRNA levels are shown as a heat map. Hierarchical clustering of aberrantly expressed miRNAs with significantly different expression was performed using Sabiosciences’ online data analysis tool. **Figure** **S3.** Identification of potential target genes for exosomal miRNAs. Venn diagrams showing the intersection between the predicted target genes of plasma exosomal miRNAs from TargetScan and miRDB, which provide a list of predicted miRNA target genes according to different algorithms. **Figure** **S4.** Characterization of exosomes in plasma of sepsis patients and healthy controls by flow cytometry. The graph depicts the percentage of positive events of 50,000 vesicles. Exosomes from sepsis patients and healthy controls were incubated with CD63 (exosome marker) and CD 11b (monocyte marker). **Figure** **S5.** Quantitative RT-PCR validation for four differentially-expressed microRNAs in patients with sepsis (n= 35) and healthy controls (n = 10) from the validation cohort. **Figure** **S6.** Cluster analysis of 405 predicted genes using STRING. Using miRsystem database, a total of 1817 target genes were identified for the four-miRNAs. Tabulated results of enriched pathways of miRNA target genes based on the consistency across multiple algorithms and observed/expected ratios. The analysis parameters in STRING were as follows: hit frequency = 3, observed to expected ratio ≥1, and matched pathways from the Kyoto Encyclopedia of Genes and Genomes database. The 934 target genes were entered into the STRING database to construct a protein–protein interaction (PPI) network, which shows the physical and functional interactions among genes. PPI networks were constructed based on important susceptibility genes. The networks were evaluated based on two topological parameters, combined scores, and degree. Degree ≥7 and a combined score ≥0.9 were cut-off criteria. Dots represent genes and lines represent interactions. **Figure** **S7.** KEGG pathway ranking for predicted target genes of four grouped miRNAs using miRSystem (Rank score ≥ 1). **Figure** **S8.** Receiver operating characteristic curves of the predictive value of four miRNAs for in-hospital mortality in patients with sepsis from the validation cohort. **Figure** **S9.** Quantitative RT-PCR validation for four differentially-expressed microRNAs in sepsis patients who had no comorbidities (n = 24) and healthy controls (n = 11) from the discovery cohort.

**Additional** **file** **2:** Quantity and quality of the exosomal RNA. Details are described in the Excel file.

## Data Availability

The data that support the findings of this study are available on request from the corresponding author. The data are not publicly available due to privacy or ethical restrictions.

## Abbreviations

APACHE II: Acute Physiology and Chronic Health Evaluation II
AUC: Area under the curve
CRP: C-reactive protein
DAVID: Database for Annotation, Visualization and Integrated Discovery
EV: Extracellular vesicle
FoxO: Forkhead box protein O
GO: Gene Ontology
ICU: Intensive care unit
IQR: Interquartile range
KEGG: Kyoto Encyclopedia of Genes and Genomes
MAPK: Mitogen-activated protein kinase
miRNA: MicroRNA
mTOR: Mammalian target of rapamycin
PI3K-Akt: Phosphatidylinositol 3-kinase/protein kinase B
PCT: Procalcitonin
PPI: Protein–protein interaction
ROC: Receiver operating characteristic
SAPS 3: Simplified Acute Physiology Score 3
SOFA: Sequential Organ Failure Assessment
