# Supplementary material for: Prognostic implication of downregulated exosomal miRNAs in patients with sepsis: a cross-sectional study with bioinformatics analysis
Source: J Intensive Care. 2023 Aug 3;11:35. doi: 10.1186/s40560-023-00683-2 (PMC10399058; doi:10.1186/s40560-023-00683-2)

**Additional file 1.**

**Prognostic implication of downregulated exosomal miRNAs in patients with sepsis**

Beomsu Shin, Jin Young Lee, Yunjoo Im, Hongseok Yoo, Junseon Park, Joo Sang Lee, Ki-Young Lee, Kyeongman Jeon

**Table S1**. Demographic and clinical characteristics of the validation cohort with sepsis (N = 35)

|  | Number (%) of patients or median (IQR) |
| --- | --- |
| Age, years | 64 (52–72) |
| Sex, male | 27 (77.1) |
| BMI, kg/m^2^ | 23.0 (20.6–26.1) |
| Comorbidity  Malignancy  Diabetes  Chronic obstructive pulmonary disease  Chronic kidney disease  Cerebrovascular disease  Chronic liver disease  Congestive heart failure  Connective tissue disease | 17 (48.6)  10 (28.6)  5 (14.3)  6 (17.1)  1 (2.9)  2 (5.7)  2 (5.7)  2 (5.7) |
| Clinical Status at ICU admission  Need for mechanic ventilation  Need for vasopressor support | 18 (51.4)  18 (51.4) |
| Severity of Illness  SAPS3 score  APACHE II score  SOFA score | 52 (45–63)  22 (20–38)  8 (7–11) |
| Laboratory findings  CRP, mg/dL  Lactic acid, mmol/L  Procalcitonin, ng/mL  IL-6, pg/mL  Platelet count, 10³/μL  Albumin, g/dL  Total bilirubin, mg/dL  Creatinine, mg/dL | 13.2 (6.5–25.1)  2.4 (1.8–4.0)  2.92 (0.75–15.83)  Not available  130 (76–177)  2.9 (2.5–3.2)  0.9 (0.6–1.7)  1.2 (0.9–2.0) |

IQR, interquartile range; BMI, body mass index; ICU, intensive care unit; SAPS 3, simplified acute physiology score 3; APACHE II, acute physiology, and chronic health evaluation II; SOFA, sequential organ failure assessment; CRP, C-reactive protein; IL-6, interleukin-6.

**Figure S1.** Bioanalyzer exosomal RNA quality control data. An ANA Agilent 2100 Bioanalyzer was used to examine exosomal RNA quality (Agilent Technologies, Inc. Santa Clara, CA, USA). The RNA ladder standard (in the first lane) contains six RNA fragments ranging between 0.2–6 kb. Representative bands of our sample’s RNA (in the second lane) showing 5S (120 nt), 18S (1,900 nt), and 28S rRNA (4,700 nt). For exoRNA bands, all samples showed an obvious band in the small RNA area.


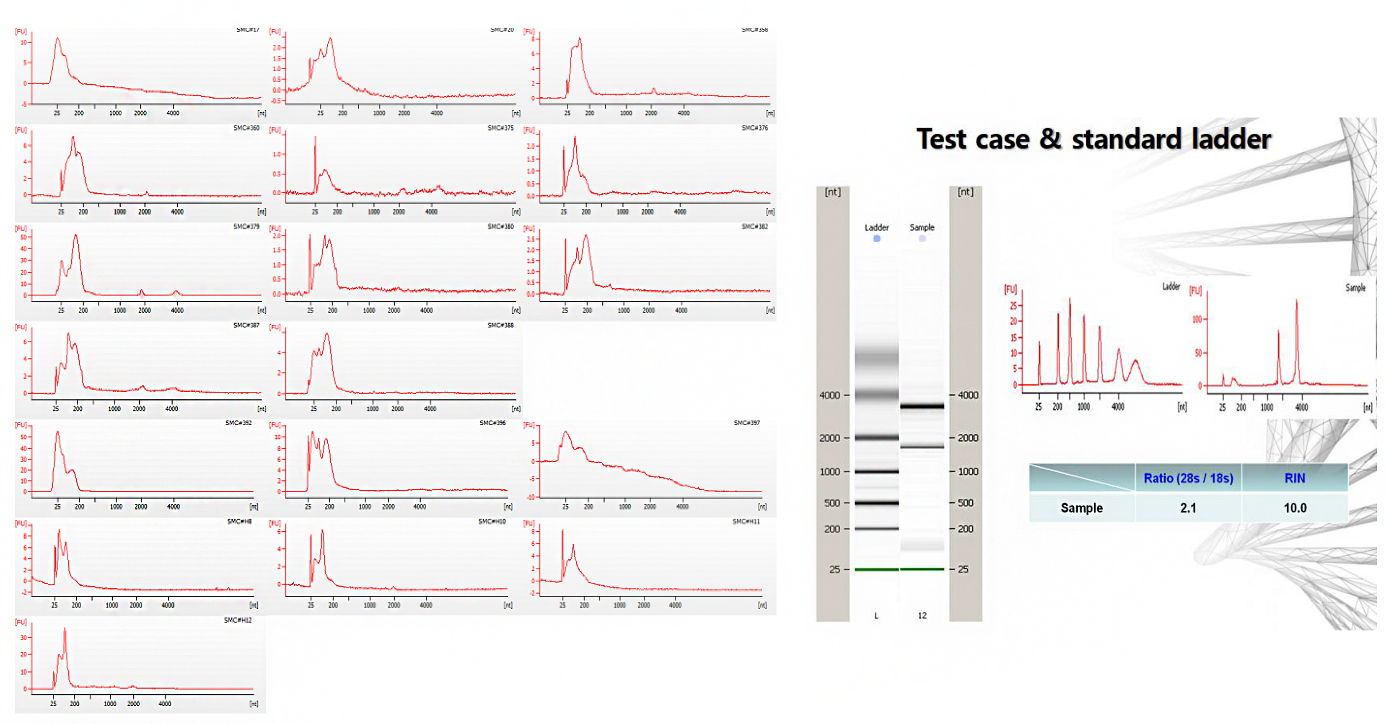


**Figure S2.** Hierarchical clustering of exosomal miRNA expression in a selected group of 135 sepsis patients utilizing 179 differentially-expressed miRNAs. Exosomal miRNA levels are shown as a heat map. Hierarchical clustering of aberrantly expressed miRNAs with significantly different expression was performed using Sabiosciences’ online data analysis tool.


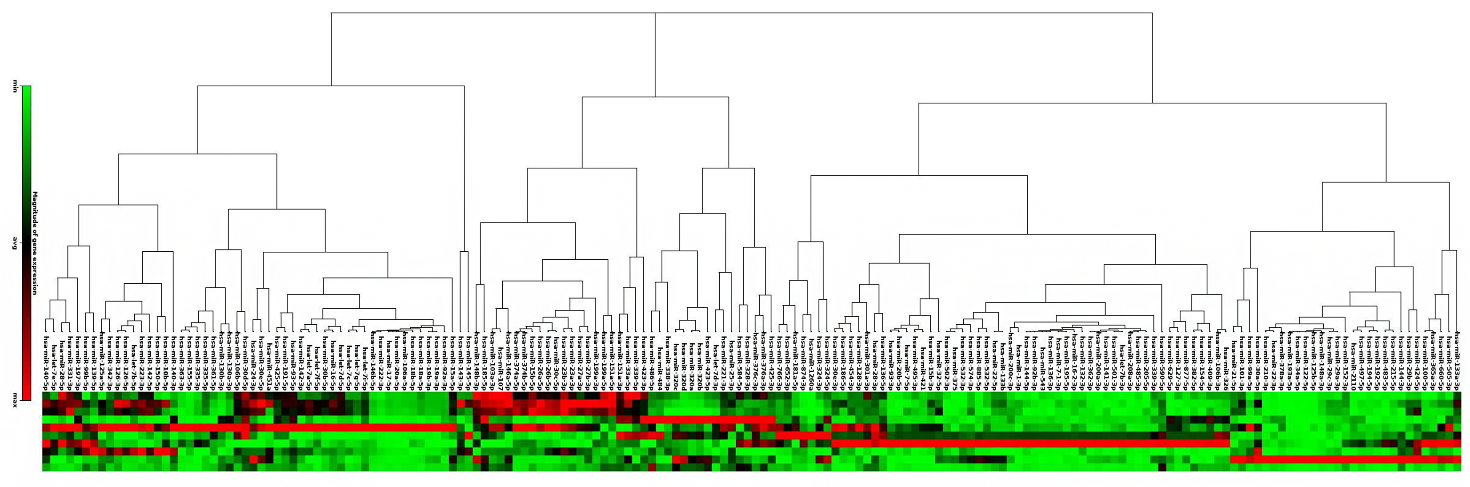


**Figure S3.** Identification of potential target genes for exosomal miRNAs. Venn diagrams showing the intersection between the predicted target genes of plasma exosomal miRNAs from TargetScan and miRDB, which provide a list of predicted miRNA target genes according to different algorithms.


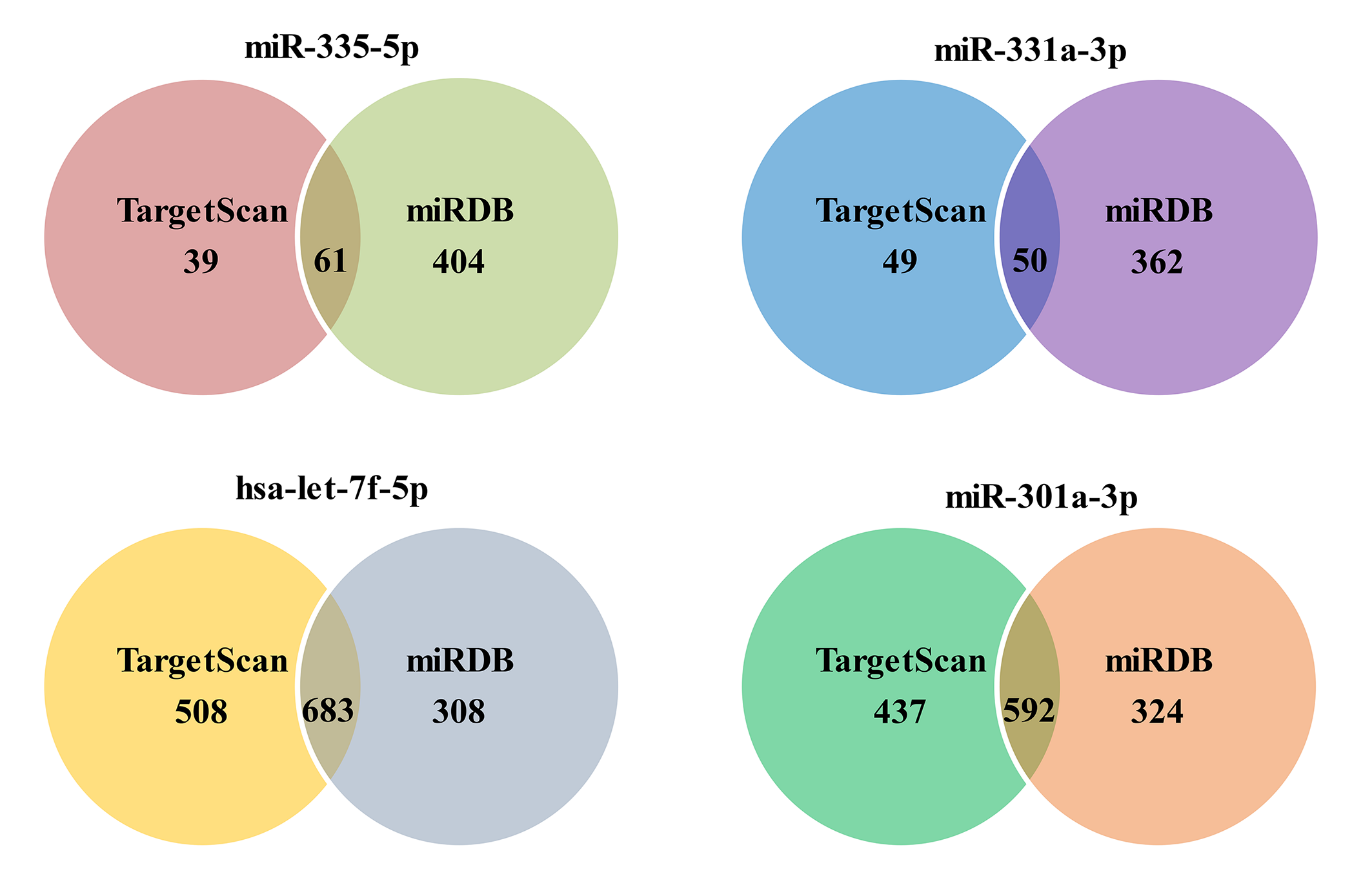


**Figure S4.** Characterization of exosomes in plasma of sepsis patients and healthy controls by flow cytometry. The graph depicts the percentage of positive events of 50,000 vesicles. Exosomes from sepsis patients and healthy controls were incubated with CD63 (exosome marker) and CD 11b (monocyte marker).

**

**

**Figure S5.** Quantitative RT-PCR validation for four differentially-expressed microRNAs in patients with sepsis (n= 35) and healthy controls (n = 10) from the validation cohort.


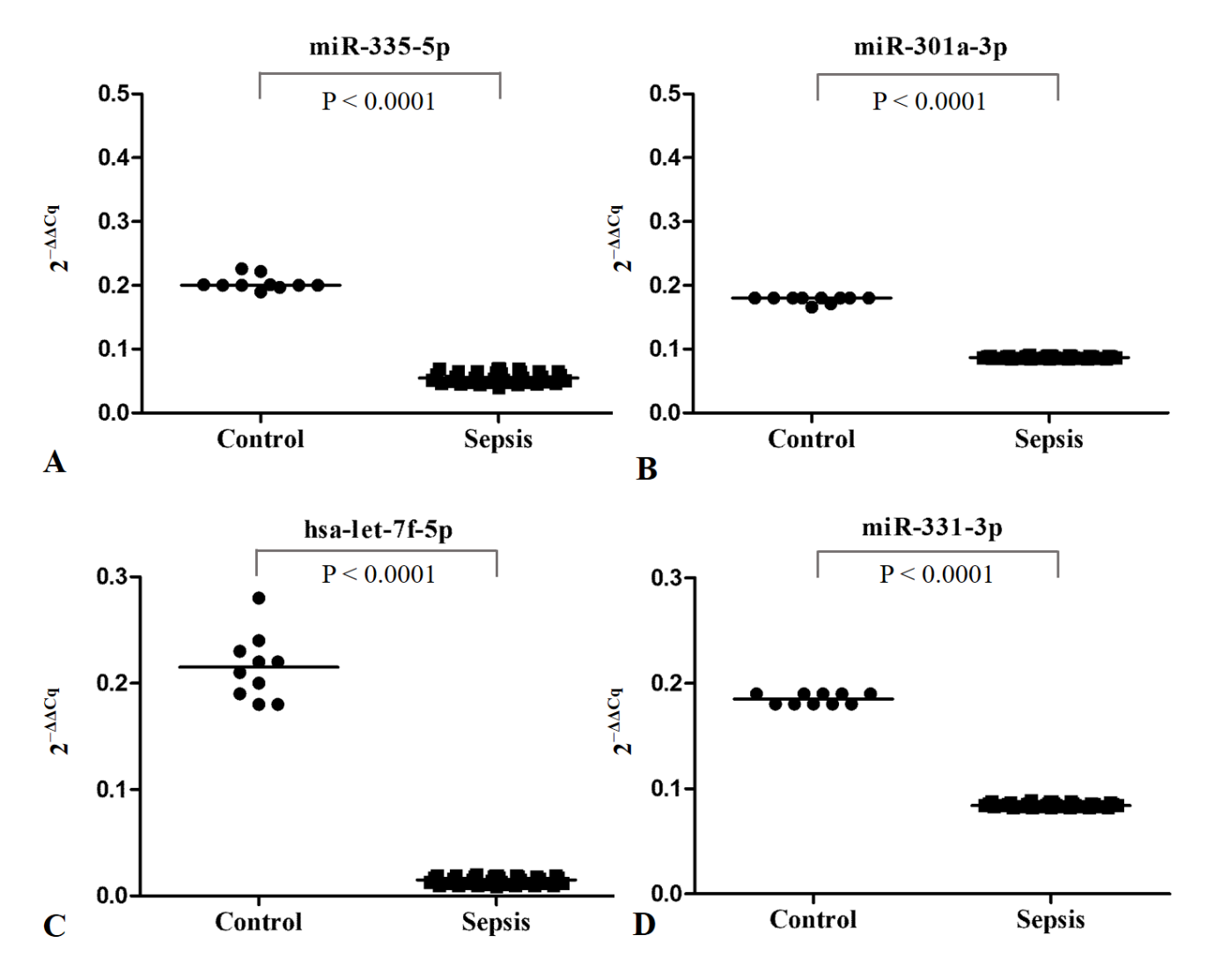


**Figure S6.** Cluster analysis of 405 predicted genes using STRING. Using miRsystem database, a total of 1817 target genes were identified for the four-miRNAs. Tabulated results of enriched pathways of miRNA target genes based on the consistency across multiple algorithms and observed/expected ratios. The analysis parameters in STRING were as follows: hit frequency = 3, observed to expected ratio ≥1, and matched pathways from the Kyoto Encyclopedia of Genes and Genomes database. The 934 target genes were entered into the STRING database to construct a protein–protein interaction (PPI) network, which shows the physical and functional interactions among genes. PPI networks were constructed based on important susceptibility genes. The networks were evaluated based on two topological parameters, combined scores, and degree. Degree ≥7 and a combined score ≥0.9 were cut-off criteria. Dots represent genes and lines represent interactions.


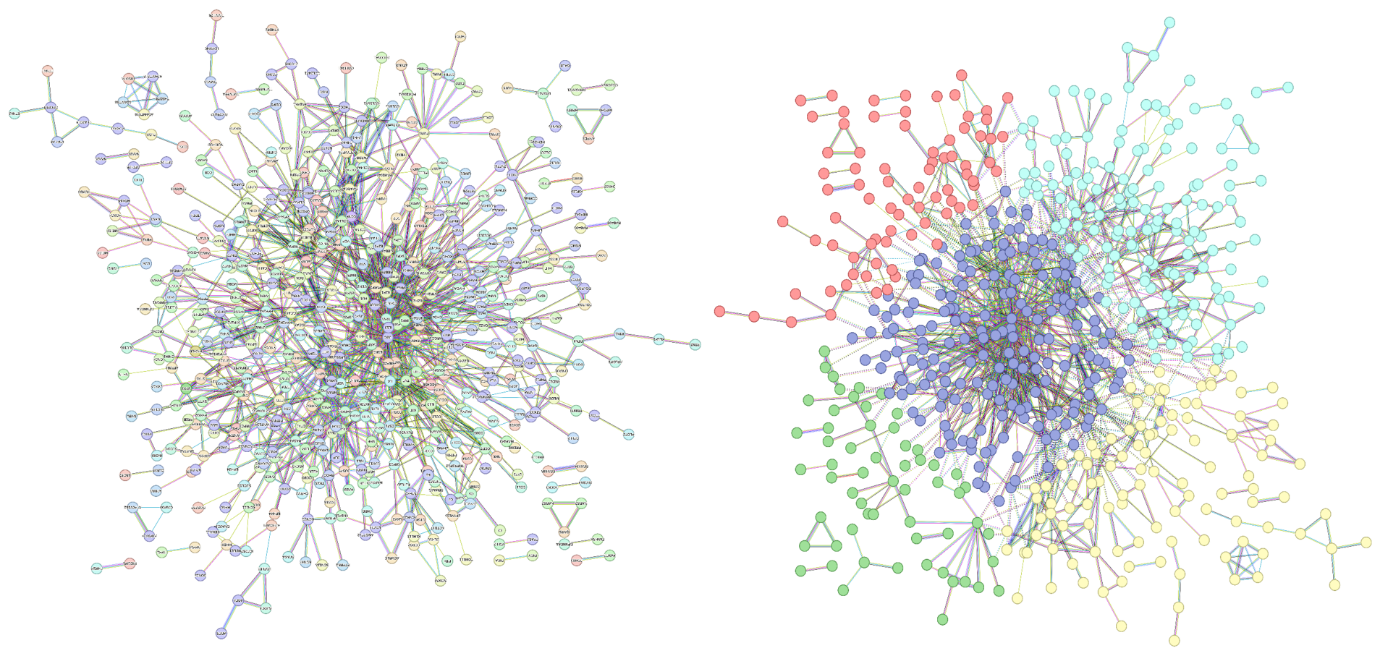


**Figure S7.** KEGG pathway ranking for predicted target genes of four grouped miRNAs using miRSystem (Rank score ≥ 1).





**Figure S8.** Receiver operating characteristic curves of the predictive value of four miRNAs for in-hospital mortality in patients with sepsis from the validation cohort.





**Figure S9.** Quantitative RT-PCR validation for four differentially-expressed microRNAs in sepsis patients who had no comorbidities (n = 24) and healthy controls (n = 11) from the discovery cohort.


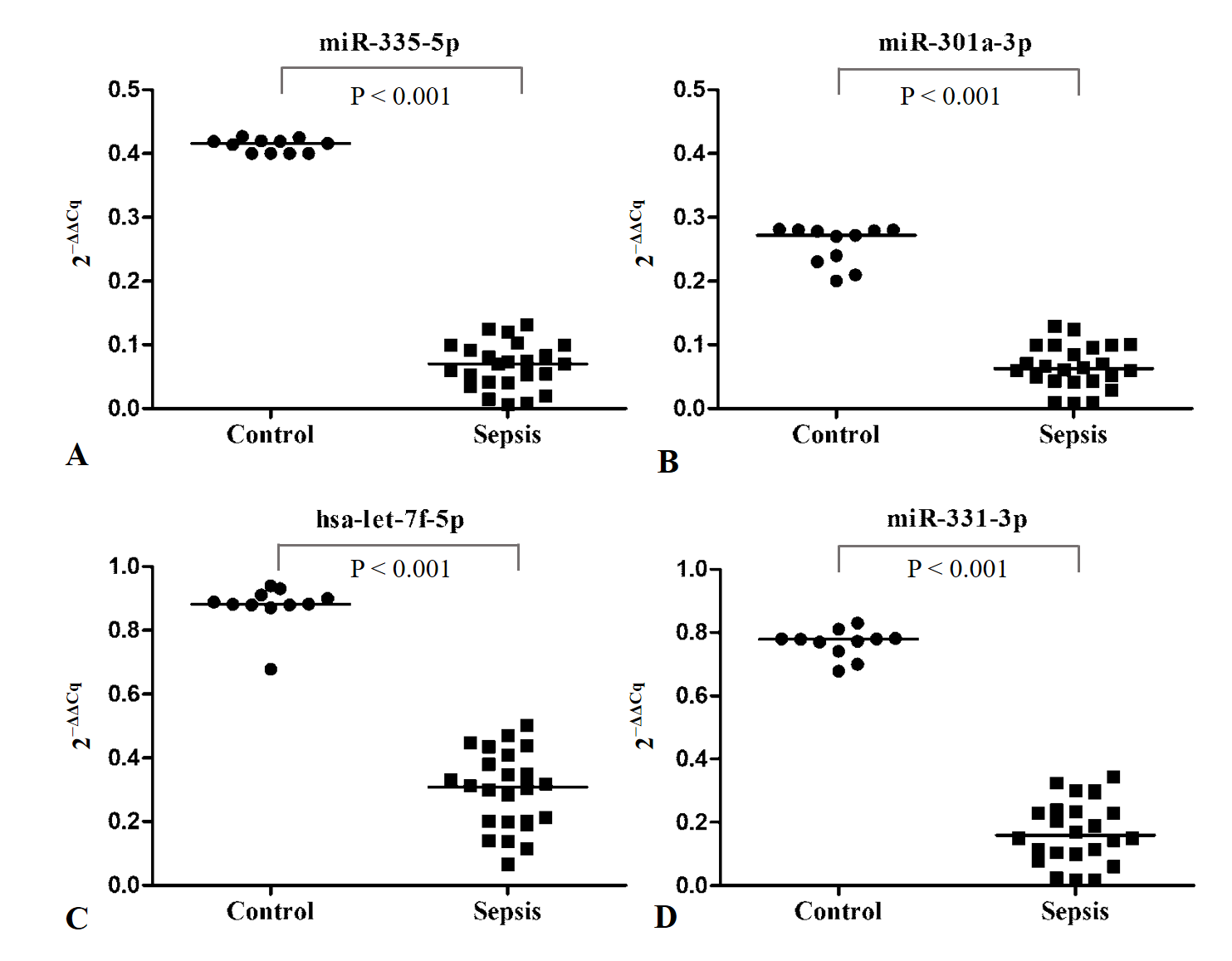

Supplement: Supplementary file 1 — Additional file 1: Table S1. Demographic and clinical characteristics of the validation cohort with sepsis (N = 35). Figure S1. Bioanalyzer exosomal RNA quality control data. An ANA Agilent 2100 Bioanalyzer was used to examine exosomal RNA quality (Agilent Technologies, Inc. Santa Clara, CA, USA). The RNA ladder standard (in the first lane) contains six RNA fragments ranging between 0.2–6 kb. Representative bands of our sample’s RNA (in the second lane) showing 5S (120 nt), 18S (1,900 nt), and 28S rRNA (4,700 nt). For exoRNA bands, all samples showed an obvious band in the small RNA area. Figure S2. Hierarchical clustering of exosomal miRNA expression in a selected group of 135 sepsis patients utilizing 179 differentially-expressed miRNAs. Exosomal miRNA levels are shown as a heat map. Hierarchical clustering of aberrantly expressed miRNAs with significantly different expression was performed using Sabiosciences’ online data analysis tool. Figure S3. Identification of potential target genes for exosomal miRNAs. Venn diagrams showing the intersection between the predicted target genes of plasma exosomal miRNAs from TargetScan and miRDB, which provide a list of predicted miRNA target genes according to different algorithms. Figure S4. Characterization of exosomes in plasma of sepsis patients and healthy controls by flow cytometry. The graph depicts the percentage of positive events of 50,000 vesicles. Exosomes from sepsis patients and healthy controls were incubated with CD63 (exosome marker) and CD 11b (monocyte marker). Figure S5. Quantitative RT-PCR validation for four differentially-expressed microRNAs in patients with sepsis (n= 35) and healthy controls (n = 10) from the validation cohort. Figure S6. Cluster analysis of 405 predicted genes using STRING. Using miRsystem database, a total of 1817 target genes were identified for the four-miRNAs. Tabulated results of enriched pathways of miRNA target genes based on the consistency across multiple algorithms and obse [file 40560_2023_683_MOESM1_ESM.docx]
